# Abastracts/Sommaires/Zusammenfassungen

**DOI:** 10.1155/S146392468000003X

**Published:** 1980


### Abstracts Sommaires Zusammenfassungen

### Page 11

**Automatic analysis: the laboratory manager's problems**

**J. K. Foreman**

This paper summarises the problems that may be encountered when introducing automation into an analytical laboratory. The author provides a generic guide and a logical pattern for discussion of the responsibilites of the laboratory manager.

**Analyze automatique: Les problèmes du chef de laboratoire**

Ce travail résume les problèmes qui se présentent à l'introduction de l'automation dans le laboratoire analytique. L'auteur donne des directives ainsi qu'un procédé logique pour la discussion des responsabilités du chef de laboratoire.

**Automatische Analyse: Die Probleme des Laborleiters**

Die Arbeit fasst die Probleme zusammen, die bei der Einführung der Automation ins analytische Laboratorium auftreten. Der Autor gibt dazu eine Aneleitung und eine logische Aufschlüsselung zur Diskussion der Verantwortlichkeiten des Laborleiters.

### Page 15

**A semi-automatic system for subsampling heterogeneous foods**

**J. F. Brown and H. T. Slover**

Nutrient analysis of complex foods requires that representative subsamples be obtained from homogeneous slurries. A low cost system has been developed that is superior to manual techniques in speed and precision. Components of the system are essentially a modified Omni-Mixer, an easily manufactured dispenser tip and simple electronic controls. Slurry viscosity is measured as a function of mixer RPM and controlled by solvent volume and temperature regulation. Following homogenization, a continuous flow of slurried sample is established by inert gas pressure from which discrete aliquots are taken; mixing is continued while samples are being dispensed. Multiple subsamples as small as 0.2 ml may be dispensed at the rate of 1 ml per second. Cleaning is accomplished without disassembly.

**Un système semiautomatique pour la prise de subéchantillons dans des produits alimentaires hétérogènes**

L'analyse des valeurs nutritives de produits alimentaires complexes demande la prise de subéchantillons représentatifs de bouillies homogènes. Un système bon marché plus rapide et précis que les méthodes manuelles a été développé. Le système consiste esentiellement d'un Omni-Mixer modifié, d'un pointe de dosage facile à réaliser et d'un contrôle électronique simple. La viscosité de la bouillie est mesurée en fonction de la vitesse de rotation du mélangeur et adjustée à l'aide de solvents et de la temperature. Après la homogénéisation une pression est appliqué avec un gas inerte produisant un débit continu de bouillie, qui permet la prise d'aliquots. Le mixage continue pendant la prise d'échantillons. A une vitesse de 1 ml/s des subéchantillons aussi petit que 0,2 ml peuvent être pris. Le nettoyage se fait sans démontage du système.

**Ein halbautomatisches System für die Entnahme von Unterproben aus heterogenen Nahrungsmitteln**

Die Nährwertanalyse komplexer Nahrungsmittel erfordert die Entnahme repräsentativer Unterproben von homogenen Breien. Es wurde dazu ein billiges System entwickelt, das manuellen Techniken in Geschwindigkeit und Präzision überlegen ist. Das System besteht im wesentlichen aus einem modifizierten Omni-Mixer, einer leicht herstellbaren Dosierspitze und einer einfachen elektronischen Steuerung. Die Viskosität des Breis wird in Funktion der Umdrehgeschwindigkeit des Mixers gemessen und mit Hilfe von Lösungsmittelzugabe und Temperaturregelung gesteuert. Nach der Homogenisierung wird mit Inertgas unter Druck ein kontinuierlicher Fluss von breiiger Probe erzeugt, dem einzelne Aliquote entnommen werden; das Mixen geht weiter, während Proben ausgegeben werden. Vielfache Unterproben bis hinab zu 0.2 ml Volumen können bei einer Geschwindigkeit von 1 ml pro Sekunde ausgegeben werden. Die Reinigung erfolgt ohne Demontage des Systems.

### Page 19

**The use of a simple 8 bit microprocessor as a flexible sequence controller for developing laboratory automation**

**F. Morley**

When developing automatic systems for analytical procedures, the designer requires a sequence controller that allows quick and easy changes to the parameters. This situation is not readily achieved with conventional methods of control (i.e. cam timers). However, the introduction of inexpensive microprocessor systems allows the construction of sequence controllers having all the flexibility required, and also the possibility of providing more complete system control.

**Utilisation d'un simple microprocesseur a 8 bit pour le contrôle sequentiel flexible dans le développement d'automation de laboratoire**

Dans le développement de systèmes automatiques pour des procédures analytiques, le constructeur a besoin de controlleurs de séquences qui permettent un changement rapide et facile de paramètres. Avec les moyens conventionels (p.ex. minuterie de contact) ceci n'est pas facile. Par l'introduction de systèmes a microprocesseur bon marchés la réalisation de controlleurs de séquence avec la flexibilité requise est rendue possible et permet un contrôle de système plus complet.

**Verwendung eines einfachen 8-Bit-Mikroprozessors als flexibler Schrittgeber für die Entwicklung der Laborautomation**

Bei der Entwicklung automatischer Systeme für analytische Aufgaben benötigt der Konstruckteur Schrittgeber, an denen die Parameter leicht und schnell eingestellt werden können. Das wird mit konventionellen Mitteln der Steuerung (z.B. Schaltuhren) nicht leicht erreicht. Mit der Einführung billiger Mikroprozessor-Systeme wird die Realisierung von Schrittgebern ermöglicht, die die nötige Flexibilität aufweisen, und die Möglichkeit zur vollständigeren Systemsteuerung geben.

Page 22

**Decision criteria for the selection of analytical instruments used in clinical chemistry**

Discussion Papers presented at the 3rd European Congress of Clinical Chemistry, Brighton, U.K, June 1979, and at the lst South East & Asian and Pacific Congress of Clinical Biochemistry, Singapore, October 1979, Symposium convenor Prof.R.Haeckel.

Recognising the need for more information on the mechanism for selecting new clinical instrumentation, the International Federation of Clinical Chemistry Expert Panel on Instrumentation organised a short symposium. Papers were presented to discuss the basic aspects of the decision criteria. Instrument selection is an integral part of laboratory management and the laboratory infrastructure. Economics of instrumentation versus a manual approach and other aspects are considered. Once an instrumental requirement has been defined all suitable instruments will be listed. The presentation of manufacturers information according to IFCC draft proposals can then be used to aid final selection. The following aspects of the decision making criteria were then presented by the authors listed. These criteria, while appropriate to clinical chemistry, are also relevant to alternative industrial environments.

I Introduction by *R Haeckel*

II Definition of problems, types of instruments and their selection by *F L Mitchell*

III Non-monetary criteria by *H Büttner*

IV External and internal evaluation of analytical instruments in clinical laboratory sciences by *M Hjelm* and *T D Geary*

V The interaction of new instrumentation with laboratory infrastructure: modelling and simulation for planning of laboratory functions by *Bengt Sandblad*

VI Techniques for the economic evaluation of automatic analysers by *Thomas M Craig*

**Critères décisifs pour la sélection d'instruments d'analyse employés en chimie clinique**

La documentation soumise au troisième "European Congress of Clinical Chemistry" à Brighton, R.U., en juin 1979 et au premier "South East & Asian and Pacific Congress of Clinical Biochemistry", à Singapour, en octobre 1979. Organisateur du symposium: le Prof. R. Haeckel.

Dans la reconnaissance d'un besoin augmentant d'information sur le mécanisme de sélection pour les instruments cliniques, "l'International Federation of Clinical Chemistry Expert Panel on Instrumentation" vient d'organiser un bref symposium. Une documentation a été soumise, afin de permettre une discussion sur les aspects de base des critères décisifs. La sélection d'instruments represente une partie intégrale de la direction et de l'infrastructure d'un laboratoire. Le rendement de l'installation d'instruments vis-à-vis du travail manuel et autres aspects sont pris en considération. A partir du moment où le besoin d'un instrument est défini, on compose une liste des instruments qui pourraient convenir. La présentation d'une information provenant des producteurs, correspondant aux directives du IFCC, peut s'avérer utile à la sélection finale. Les aspects suivants des critères décisifs ont été presentés par les auteurs mentionnés sur la liste. Ces critères, parce qu'ils sont appropriés à la chimie clinique, sont aussi valables pour d'autres industries voisines.

I Introduction par *R Haeckel*

II Definition des problèmes, genres d' instruments et leur selection par *FL Mitchell*

III Criteres non-monétaires par *H Büttner*

IV Evaluation externe et interne d' instruments d'analyse en sciences cliniques de laboratoire par *M Hjelm* et *TD Geary*

V L'interaction entre les nouveaux instruments et l'infrastructure du laboratoire: Réalisation et simulation pour la planification de fonctions de laboratoire par *Bengt Sandblad*

VI Technique pour une evaluation économique d'analyses automatiques par *Thomas M Craig*

**Entscheidungskriterien für die Auswahl analytischer Instrumente zur Verwendung in der klinischen Chemie**

Vorträge gehalten am 3. European Congress of Clinical Chemistry, Brighton, UK, im Juni 1979, und am 1. South East & Asian and Pacific Congress of Clinical Biochemistry, Singapore, im Oktober 1979. Symposium-Organisator Prof. R. Haeckel.

In Erkenntnis des Bedarfs für mehr Information über die Entscheidungsmechanismen für die Auswahl neuer klinischer Instrumentierung hat die Fachgruppe für Instrumentierung der International Federation of Clinical Chemistry ein kurzes Symposium organisiert. Es wurden Vorträge gehalten zur Diskussion der grundlegenden Aspekte der Entscheidungskriterien. Die Auswahl von Instrumentierung stellt einen integralen Teil des Laboratorium-Managements und der Laboratoriumsinfrastruktur dar. Die Wirtschaftlichkeit von Instrumentierung im Vergleich zu einem manuellen Ansatz und anderen Aspekten wurde betrachtet. Wenn die instrumentellen Erfordernisse definiert sind, können alle geeigneten Instrumente angeführt werden. Die Darstellung der Informationen der Hersteller gemäss einem Vorschlag der IFCC kann dann zur Hilfestellung für die endgültige Auswahl verwendet werden. Die folgenden Aspekte der Entscheidungskriterien wurden daraufhin durch die unten angeführten Autoren vorgetragen. Diese Kriterien, obwohl aus der Domäne der klinischen Chemie stammend, sind auch relevant für andere industrielle Umgebungen.

I Einführung durch *R Haeckel.*

II Definition der Probleme, Typen oder Instrumente und ihre Auswahl, durch *FL Mitchell.*

III Nicht-pekuniäre Kriterien, durch *H Büttner.*

IV Externe und interne Evaluation von analytischen Instrumenten im klinischen Laboratorium, durch *M Hjelm* und *TD Geary.*

V Die Wechselwirkung zwischen neuer Instrumentierung und Laboratoriumsinfrastruktur: Modelle und Simulationen für die Planung von Laboratoriumsfunktionen, durch *Bengt Sandblad.*

VI Techniken fur die wirtschaftliche Auswertung von automatischen Analysengeräten, durch *Thomas M Craig.*

